# The use of bimetallic metal–organic frameworks as restoration materials for pollutants removal from water environment

**DOI:** 10.1098/rsos.240380

**Published:** 2024-07-31

**Authors:** Yue Yuan, Shaocong Li, Lina Zhu

**Affiliations:** ^1^ Department of Chemistry, School of Science, Tianjin University, Tianjin 300072, People’s Republic of China

**Keywords:** pollutants, wastewater treatment, synergistic effect, bimetallic organic frameworks, water environment

## Abstract

Bimetallic metal–organic frameworks (BMOFs) are a class of functional porous materials constructed by coordination between nodes containing two different metal ions and organic ligands. Studies have shown that the catalytic activity of BMOFs is greatly improved owing to the adjustment of charge distribution and the increase of active sites as well as the synergistic effect between the bimetals, and the advantages of their large specific surface area, high porosity, unique structure and dispersed active centres make them available as important organic materials applied in the field of wastewater treatment. In this review, the preparation and construction methods for BMOFs in recent years are summarized, and we focus on their removal of different types of pollutants in the aqueous environment, including ions, dyes, pharmaceuticals or personal care products, phenolic compounds and microorganisms, as well as their corresponding removal mechanisms. In addition, we provide an outlook on their future opportunities and challenges in wastewater treatment.

## 1. Introduction

Water pollution is one of the most significant environmental issues in the world [1–3]. Over the past few decades, there has been an explosion in the growth of hard-to-degrade pollutants in the environment, mainly resulting in excessive discharge of domestic sewage and industrial and agricultural wastewater, which cause secondary pollution in the surrounding natural environment. Moreover, they pose a great risk to human health at the same time [4–10].

**Figure 1.**
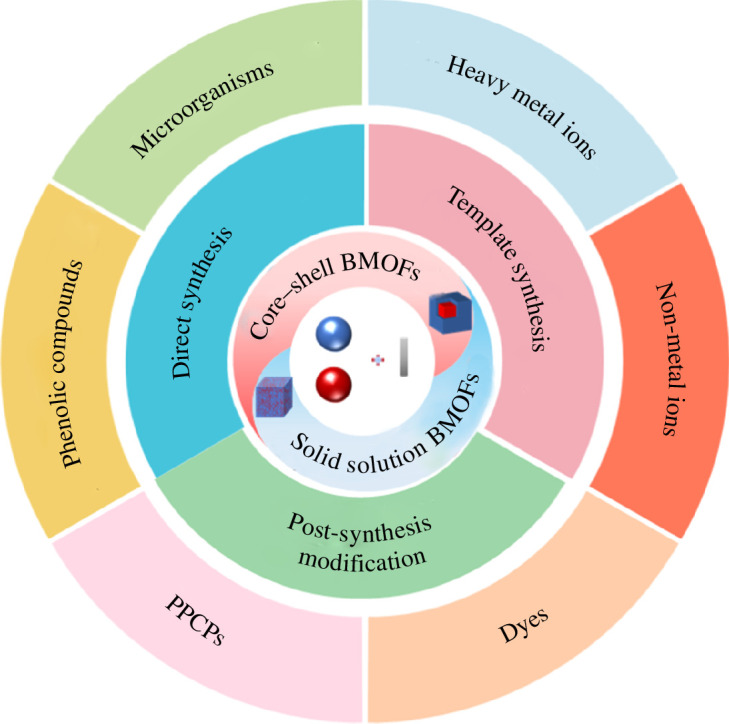
Structure and preparation of BMOFs.

Metal–organic frameworks (MOFs) are a new class of hybrid materials with a porous structure and multiple available active sites [11–13]. In order to enhance the active sites for chemical reactions, it has been proposed to construct bimetallic MOFs (BMOFs) by partially replacing the original skeleton nodes with a second metal ion [14]. Moreover, different ratios of two metal ions provide additional structural complexity and performance tenability [15]. Depending on the distribution of metal ions, BMOFs can form either solid solution or core–shell structures (figure 1). In solid solution BMOFs, the metals show a bias domain or uniform distribution throughout the crystal. In core–shell BMOFs, the MOF shell and MOF core are chemically distinct, but they are integrated into a single structure [16].

During the last decade, BMOFs have been explored extensively and exhibited high potential in removing pollutants in wastewater because of their high porosity, diverse compositions, adjustable pore structure and good stability [17]. In addition, the synergistic effect of the bimetallic sites gives the BMOFs the unique significant increase in acidity and reactivity [18,19]. BMOFs have been used to remove various organic pollutants (e.g. dyes) [20–27], pharmaceuticals or personal care products (PPCPs) [28–34] and inorganic pollutants (e.g. heavy metal ions) [17,35–39] by adsorption or photocatalysis. They can also remove organic contaminants through oxidative degradation and incomplete mineralization, named Fenton reaction or Fenton-like reactions and persulfate (PS) oxidativation. Therefore, BMOFs have become good candidates to remove pollutants in the aquatic environment.

In this review, the preparation and construction methods for BMOFs are summarized in recent years and we focus on their removal of different types of pollutants in the aqueous environment, as well as their corresponding removal mechanisms. In addition, we provide an outlook on their future opportunities and challenges in wastewater treatment.

## 2. The synthetic methods for bimetallic metal–organic frameworks

Diverse ways of preparing BMOFs have been investigated, such as direct synthesis, post-synthesis modification and template synthesis (figure 1). It is worth noting that the morphology, structure and properties of BMOFs are strongly influenced by the preparation methods, which may affect the catalytic activity of these materials when used as catalysts. Therefore, it is necessary to study the effect of the preparation method on the catalytic activity.

### 2.1. Direct synthesis

Direct synthesis is a method that mixed the desired metal ions or clusters along with the polyhedrally bridged ligands into an aqueous solution during the solvothermal synthesis [40]. This method is believed to be a one-step, green and facile fabrication technique. In this method, the reaction conditions such as the temperature of the reaction system, the type and concentration of the selected surface stabilizers and the concentration and ratio of each reactive precursor in the reaction system affect the self-assembly products [41]. Notably, the direct synthesis method is suitable for two metal ions with similar coordination ability—in other words, the simultaneous one-step synthesis of mixed MOFs with ligands [42]. The direct synthesis can be used to prepare BMOFs of both solid solution and core–shell structures.

**Figure 2.**
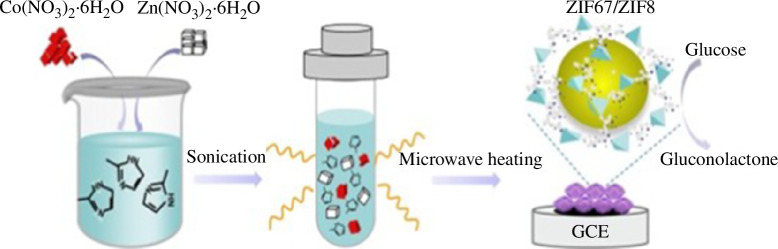
Schematic illustration showing the one-step microwave-assisted synthesis of ZIF67/ZIF8 for enzyme-free glucose detection [44].

Chen’s group dissolved different molar ratios of cobalt salt, nickel salt and polyvinylpyrrolidone (PVP) in an ethanol and water mixed solution to form solution A, and dissolved 2,5-dihydroxyterephthalic acid (DHTA) in a tetrahydrofuran (THF) solvent to form solution B [43]. The mixture of solution A and solution B was then transferred to a reactor and stored at 120°C for 24 h, to obtain the sample Co_x_Ni_1−x_–MOF-7. Notably, the central metal ion can affect the growth behaviour of MOF-74, and the morphology of MOF-74 can be effectively controlled by replacing the central ion. Li *et al*. fabricated bimetallic Co/Zn MOFs (ZIF67/ZIF8) via the microwave-assisted method [44]. The use of microwaves allowed for better preparation efficiency. Compared with the original hydrothermal or solvent-thermal method, which took hours or even days, the method took only 20 min, which greatly shortened the preparation time (figure 2).

### 2.2. Template synthesis

Generally, the template synthesis method uses the prepared precursor material as a template, and the latter material is physically or chemically embedded in the structure or loaded on the surface of the precursor material. This method is independent of the initial metal concentration in the reaction mixture; thus, the arrangement of metal ions in the MOF can be well controlled [45]. Solid solution BMOFs can be produced by this method.

**Figure 3.**
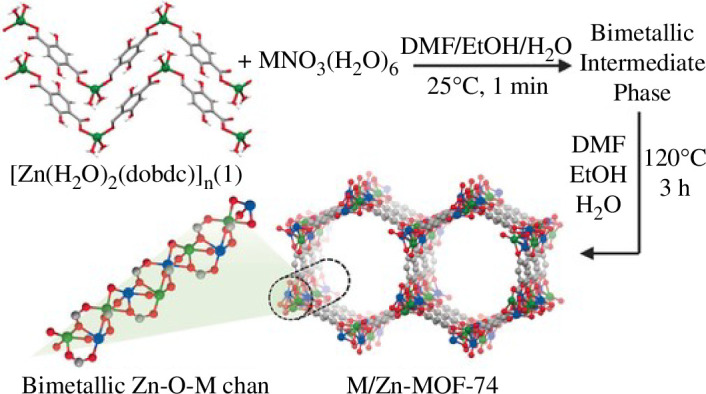
Synthetic strategy for the heterogeneous phase preparation of bimetallic MOF-74 structures via the template-directed approach, in which the one-dimensional metal–organic polymer with well-defined binding pockets was used as a structural template for the preparation of well-ordered bimetallic MOF 74 s. M = secondary metal (Mg^2+^ or Ni^2+^ = blue), Zn^2+^ = green, C = grey and O = red. Hydrogen atoms are omitted for clarity [47].

Geary *et al*. synthesized two different cross-linked ligand dimers containing heat-resistant tertiary carbamate bonds for cleaving into aryl and alkyl amines that acted as structural templates with precursors [46]. They found that the organic amines had good binding sites to coordinate various metals (e.g. Mn(II), Fe(II), Co(II), Ni(II), Cu(I) and Cu(II)) to form bimetallic iminopyridine and bis(2-pyridinylmethyl)amine complexes. Kim *et al*. employed a one-dimensional metal–organic polymer containing 2,5-dihydroxy-1,6-benzenedicarboxylate and zinc ions as a structural template and precursor for the synthesis of bimetallic MOF-74 [47]. Because of well-defined binding sites in the polymer structure, the precise coordination with secondary metal ions (Mg^2+^ or Ni^2+^) can be realized. Thermal treatment of this metal-impregnated polymer in a dimethylformamide (DMF)/ethanol (EtOH)/H_2_O solvent mixture resulted in the formation of a bimetallic MOF-74 structure. It is worth noting that regardless of the initial stoichiometry of the two ions in the reaction mixture, a bimetallic MOF-74 structure with a metal ratio close to 1 : 1 can be formed. Generally, ionic and neutral surfactants or block copolymers were used as templates to direct the formation of mesopores inside the MOF crystals for the preparation of stabilized MOFs with controllable porosity, where the porosity of the MOFs can be adjusted by controlling the amount of the template (figure 3).

### 2.3. Post-synthesis modification

Post-synthesis modification method refers to a method of chemical conversion or exchange on pre-synthesized MOF materials; some BMOFs that cannot be synthesized directly can be synthesized before obtaining the target product through a metal ion exchange process [48]. In this process, the extent, rate and reversibility of the exchange process are influenced by the coordination number and environment of the secondary building unit, the ionic radius and valence state of the metal ion, the MOF lattice and the solvent. Because of unique properties of small molecular size and relatively high ligand field strengths, methanol is the most broadly adopted solvent in the polyol technique in post-synthesis modification [49]. Solid solution BMOFs and core–shell BMOFs can be produced by this method.

**Figure 4.**
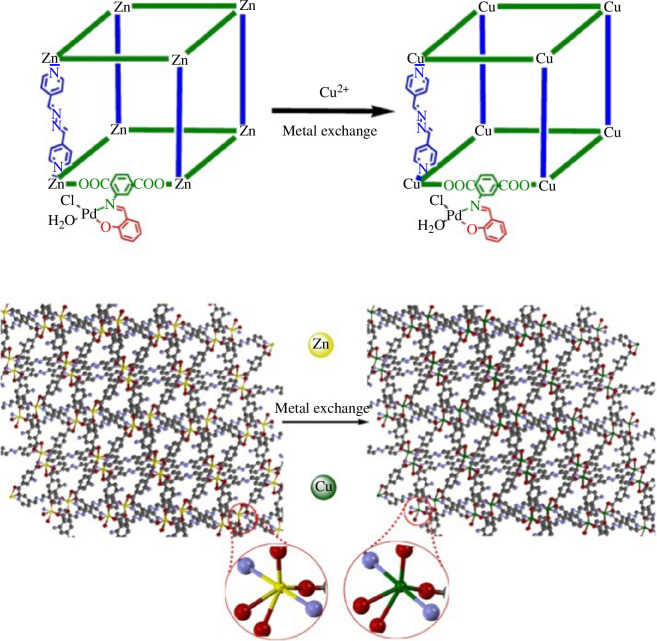
Preparation of the Cu-MOF-[Pd] by inorganic post-synthetic modification [50].

Salahshournia *et al*. synthesized a kind of bimetallic MOF (Cu-MOF-[Pd]) via post-synthesis modification [50]. In this research, the authors used Zn-MOF (TMU-17-NH_2_) to form grafted salicylaldehyde via Schiff base reaction, and complexes with Pd(II) were formed. Then, based on the synthesized MOF (Zn-MOF-[Pd]), Cu-MOF-[Pd] was obtained by the metal exchange method. Comparison of the Fourier-transform infrared (FT-IR) spectra and powder X-ray diffraction (PXRD) plots of Zn-MOF-[Pd] and Cu-MOF-[Pd] showed that the structure remained stable after metal exchange and the integrity of the backbone was not affected (figure 4). In addition, Song *et al*. further demonstrated that Zn-HKUST-1 and Zn-PMOF-2 immersed in an methanol solution of Cu(II) can be converted into Cu-based MOFs with thermodynamically more stable homogeneous structures by ion exchange, and the conversion was not reversible [51]. The results showed that complete metallization can be achieved in the Zn-PMOF-2 framework system, while only partial metallization can be accomplished in the Zn-HKUST-1 framework system owing to the flexibility of the skeleton metal centre.

As mentioned above, solid solution BMOFs can be constructed by direct synthesis, post-synthesis modification method and template synthesis, while core–shell BMOFs can be produced by direct synthesis and post-synthesis modification method. Thanks to the variety of organic ligands and metal centres as building blocks, BMOFs and their composites offer an excellent tool for studies. Based on the successful studies in recent years, we believe that BMOF-based composites would have a great development in future applications of wastewater treatment.

## 3. Removal of various pollutants

Pollutants are particles that cause environmental damage by getting into the environment naturally or through humankind [52]. Many studies have shown that BMOFs as efficient reducing agents or catalysts can remove pollutants such as ions [35–38,53–56], dyes [20–27], PPCPs [28–34], phenolic compounds [57,58] and microorganisms [59]. Table 1 summarizes the removal of different kinds of pollutants by some of the currently reported BMOFs.

**Table 1.** Removal of different pollutants by BMOFs.

pollutant		BMOFs	removal mechanism	removal effect	ref.
heavy metal ions	Pb(II)	Fe_0.05_HKUST-1	adsorption	*Q*_max_ = 564.9 mg g^−1^	[35]
		Ni/Cd-MOF	adsorption	*Q*_max_ = 950.61 mg g^−1^	[17]
	Sb(III)	Zr_0.8_Fe_0.2_-MOF-808	adsorption	*Q*_max_ = 310 mg g^−1^	[36]
	Sb(V)			*Q*_max_ = 524 mg g^−1^	
	Cd(II)	Ag/Fe MOF	adsorption	*Q*_max_ = 265 mg g^−1^	[37]
	Pb(II)	CdK-m-COTTTB	adsorption	*Q*_max_ = 636.94 mg g^−1^	[38]
	Tb(III)			*Q*_max_ = 432.90 mg g^−1^	
	Zr(IV)			*Q*_max_ = 357.14 mg g^−1^	
	U(VI)	MgAl-DHBDC/LDH	adsorption	*Q*_max_ = 133.76 mg g^−1^	[39]
non-metal ions	As(III)	aFMM-120	adsorption	*Q*_max_ = 161.6 mg g^−1^	[53]
	As(V)	Fe/Mg-MIL-88B	adsorption	*Q*_max_ = 303.6 mg g^−1^	[54]
	Se(IV)	UiO-66(Fe/Zr)	adsorption	*Q*_max_ = 196 mg g^−1^	[55]
	Se(VI)			*Q*_max_ = 258 mg g^−1^	
	P(V)	La/Ca-BDC-1	adsorption	removal = 90%	[56]
dyes	MB	HNU-29	photocatalysis	removal = 92%	[20]
		FeCo-BDC	persulfate activation	removal = 100%	[21]
		FeNi_1/60_-BDC	Fenton-like reaction	removal = 100%	[22]
	RhB	Zn/Zr-MOF-1	photocatalysis	removal = 97.4%	[23]
		Fe/Ni-MOF/CCAC	photocatalysis	removal = 100%	[24]
		10%Ni/Fe-MOF	Fenton-like reaction	removal = 96%	[25]
		MIL-53(Fe,Ni)	persulfate activation	removal = 93.9%	[26]
	AO7	Fe_4_Mn_6_-Fc-MOFs	persulfate activation	removal = 92%	[27]
PPCPs	CIP	CUMSs/MIL-101(Fe,Cu)	Fenton-like reaction	removal = 100%	[28]
		MIL-101(Fe,Co)	Fenton-like reaction	removal = 97.8%	[29]
	TC	FeNi-LDH/BMNSs	Fenton-like reaction	removal = 95.76%	[30]
	NOR	FeCo-MOF-2	photocatalysis	removal = 99.1%	[31]
	SMX	Fe_0.75_Cu_0.25_(BDC)	Fenton-like reaction	removal = 100%	[32]
	DC	Zr/Fe-MOFs	adsorption	removal = 87.5%	[33]
	cefradine	UiO-67-Co	adsorption	*Q*_max_ = 1091 mg g^−1^	[34]
phenolic compounds	2,4-DCP	Fe-Cu-MOF@C	persulfate activation	removal = 99.4%	[57]
	phenol	Ag_3_PO_4_@UMOFNs	photocatalysis	removal = 100%	[58]
	bisphenol A			removal = 98.9%	
microorganisms	*E. coli*	Co_x_Cu_y_-CAT-1	photocatalytic–photothermal effect	bacterial mineralization rate = 70%	[59]

### 3.1. Heavy metal ions

Heavy metal ions are metal ions characterized by bioaccumulation, carcinogenicity, non-biodegradability, high toxicity and environmental pollution. They are widely used in industries such as metal plating, mining, pharmaceuticals and pest control. It is well known that exposure to water containing heavy metals such as lead, cadmium and copper can cause serious illnesses, for example, lung, immune and endocrine disruption and cancer in humans [60].

In recent years, some studies are focused on the adsorption of different heavy metal ions by BMOFs, and it is shown that they can significantly improve the removal of heavy metal ions compared with monometallic MOFs. During the past 10 years, the removal of multiple heavy metal ions has attracted many researchers’ attention. El-Yazeed *et al*. developed Ag/Fe-MOFs with the ability of high adsorption capacity and adsorption rate for Cd(II) and Cu(II) and found that the adsorption capacity reached 265 and 213 mg g^−1^, respectively [37]. Shen *et al*. designed a new BMOF of removing both hard and soft metal ions (CdK-m-COTTTB) using sulfur-rich *m*-cyclooctatetrathiophene-tetrabenzoate (*m*-H_4_COTTTB) as the organic ligand and oxygen-rich bimetallic clusters as the inorganic nodes [38]. The adsorption of Pb^2+^, Tb^3+^ and Zr^4+^ ions is up to 636.94, 432.90 and 357.14 mg g^−1^, respectively. Moreover, the absorbents showed the capability to remove more than 12 different metal ions in both separate and multi-component solutions. The result exhibited that this material had comparable absorption capacity to specific MOFs.

As a renewable energy source, nuclear energy makes an important contribution to meeting the global need for sustainable, low-carbon and reliable energy. However, the operation of nuclear power station and other anthropogenic causes can result in the uncontrolled introduction of radioactive waste into the environment [61,62]. Uranium is a representative common type of nuclear-contaminated waste. Because of the toxicity and radioactivity of uranium, the presence of highly enriched uranium in groundwater and surface water can seriously contaminate the environment and even extend to the entire biological chain, leading to a threat to the health of living organisms. Recently, to meet the demand for purified water, various materials have been explored for the removal of uranium from the water environment, such as inorganic materials (e.g. LDH and MS materials and metal oxides) and organic polymers (e.g. resins, cellulose and chitosan). Owing to the functionalization of MOFs, they can introduce open metal sites and functional groups into the structure. Thus, MOF-based adsorbents are an ideal tool for uranium removal [63,64]. Li *et al*. reported a kind of MgAl-DHBDC/LDH composite by etching MgAl BMOF template. This material exhibited excellent removal effect for U(VI); under pH = 4, the removal rate of U(VI) was 96.64%, and the removal rate remained above 90% after four cycles [39].

### 3.2. Non-metal ions

Based on a recent study, the excessive intake of non-metal ions such as selenium (Se) and arsenic (As) can have a lethal effect on human health; for example, it can induce cardiovascular, immunological and neurological dysfunctions [65]. Therefore, the water contamination by non-metallic ions should also be given sufficient attention. Relying on the synergistic effect of bimetals and the unique porous structure, BMOFs have excellent properties such as large capacity, fast kinetic properties, good immobilization ability and water stability, thus they can be used as novel and efficient adsorbent materials for the remediation of inorganic non-metallic ions in the aqueous environment.

Zhang *et al*. reported an amorphous FeMn-MOF-74 adsorbent called aFMM-120 for the efficient remediation of arsenic-contaminated water, which was synthesized by a simple temperature-controlled crystallization method [53]. The morphology and crystalline phase of the synthesized aFMM-120 were analysed by transmission electron microscopy (TEM) and XRD, and it was shown that the surface morphology of the material was an individual marigold-like microparticle with a uniform diameter distribution of about 3 mm and no crystallinity. Under neutral conditions, the saturated arsenic uptake of aFMM-120 adsorbent with an Fe/Mn ratio of 1.96 could reach 161.6 mg g^−1^, with a significantly higher adsorption performance than that of any other available MOF-based adsorbent. Guo *et al*. constructed a kind of BMOF called UiO-66(Fe/Zr) for the removal of selenite (Se(IV)) and selenate (Se(VI)) from the aqueous environment [55]. Because of good thermal stability, large specific surface area and uniform mesoporous structure of this material, the adsorption efficiency for selenium remained high in the pH range of 2–11, and the maximum adsorption for Se(IV) and Se(VI) was as high as 196 and 258 mg g^−1^ at pH = 3 and pH = 5, respectively. Furthermore, this adsorbent could achieve more than 99% removal of native Se(IV) and Se(VI) from 1.0 mg l^−1^ lake and tap water samples in 2.0 h.

### 3.3. Dyes

Organic dyes are one of the major components of industrial wastewater. Actually, the annual use of about 700 000−1 000 000 tons of dyes worldwide has been conservatively estimated [66]. Because of different chemical structures, the commonly used dyes can be categorized into six kinds: azo dyes, anthraquinones, indigo, triphenylmethyl, sulfur and phthalocyanine derivatives. Azo dyes are one of the most used dyes in industrial activities. Dyes can cause human disease and water pollution owing to their complex structures and persistence [67].

Feng *et al*. reported a kind of BMOF (HNU-29) that exhibited an excellent photocatalytic activity for methylene blue (MB) under visible-light irradiation without H_2_O_2_ [20]. When the catalyst dosage was 0.44 g l^−1^, the degradation rate of MB was about 97% after 100 min of visible-light irradiation. Wu *et al*. reported an iron-based MOF (FeNi_1/60_-BDC) prepared by metal doping for the removal of MB and methyl orange (MO) [22]. The authors found that Ni doping increased the specific surface area and pore volume and decreased the surface charge at the same time, which led to a significant increase in the adsorption capacity of the dyes. Compared with the corresponding monometallic MOFs, the adsorption capacity of FeNi_1/60_-BDC for MB and MO was increased by a factor of 5.3 and 2.6, respectively, which was favourable for the subsequent degradation. Qu *et al*. synthesized a series of MOFs with different Fe–Mn molar ratios (1 : 9, 2 : 8, 4 : 6 and 6 : 4) of FeMn-Fc-MOFs catalysts and selected Fe_4_Mn_6_-Fc-MOFs catalysts for the removal of Acid Orange 7 (AO7) in persulfate (PS) system [27]. Under optimal conditions, this material exhibited the removal of 92.0% for AO7 after 120 min. Electrochemical experiments demonstrated that the better catalytic performance of Fe_4_Mn_6_-Fc-MOFs than other FeMn-Fc-MOFs was owing to their stronger electron transfer ability.

### 3.4. Pharmaceuticals or personal care products

In recent decades, as the demand for medical treatment has increased and the level of medical care has developed, the emission of PPCPs is continuously increasing in the aquatic environment, posing significant threats to human health and the aquatic ecosystems [68–71]. BMOFs are considered to be a promising restorative agent for rapid and stable PPCPs removal with easy operability and low cost.

**Figure 5.**
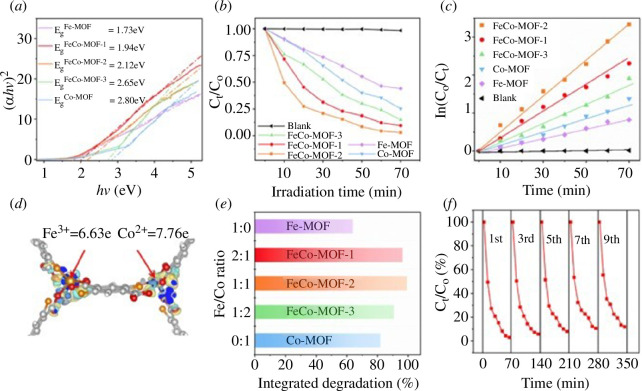
Curves of (*αhν*)^2^ versus *hν* for bandgap of MOFs from the Kubelka–Munk method (*a*); deformation charge density in FeCo-MOF-2 (*b*); degradation as a function of irradiation time for photocatalysis (*c*); degradation efficiencies of solutions after the treatment (*d*); degradation reaction kinetics of the NOR over FeCo-MOF-X (X = 1, 2, 3), Fe-MOF and Co-MOF (*e*); the cyclic performance of the photocatalytic activity of the FeCo-MOF-2 under the same conditions (*f*) [31].

Owing to the toxicity and oxidation resistance of Norfloxacin (NOR), the biodegradation degradation efficiency is often very low and it can lead to bacterial resistance [72]. Zhou *et al*. synthesized a bimetallic MOF-based photocatalyst (FeCo-MOF-2(Fe:Co = 1 : 1)) [31]. The result showed that its optical response was shifted from the UV and IR regions of the original monometallic MOFs to most of the visible region. The charge redistribution played a key role in determining the changes in electronic structure and optical response. Furthermore, FeCo-MOF-2 enabled the efficient separation of photogenerated electron–hole pairs. FeCo-MOF-2 showed an integrated degradation efficiency of 99.1% for NOR after 70 min of light irradiation with the help of improved optical properties, and the mineralization rate reached 40% within 180 min (figure 5). Tang *et al*. prepared a BMOF-based material (Fe_*x*_Cu_1−*x*_(BDC)) via a simple solvothermal method that showed excellent removal efficiency for sulfamethoxazole (SMX) when hydrogen peroxide was added to the system [32]. In this research, the Fe_0.75_Cu_0.25_(BDC) catalyst showed a complete SMX (20 mg l^−1^) removal within 120 min when the initial solution pH was 5.6, which exhibited a higher removal efficiency compared with its monometallic catalysts.

### 3.5. Phenolic compounds

Phenolic compounds are widely found in nature. In recent years, phenolic compounds have become one of the main constituents of water pollutants owing to the discharge of untreated wastewater from multiple industrial areas, such as gas, coking, oil refining and petrochemicals. Specifically, phenol, which is a popular water-soluble organic pollutant in the industries, can cause symptoms of chronic as well as acute poisoning owing to the toxic features [73].

Wang *et al*. reported the Fe-Cu-MOF@C/PS system based on the BMOFs in the degradation of 2,4-dichlorophenol (2,4-DCP) [57]. In this study, the authors found that it can reach more than 99.4% removal rate of the 2,4-DCP within 60 min. Besides, within 180 min, 83.4% of the COD removal was realized. Liang *et al*. reported the photocatalysis in the degradation of phenol and bisphenol A using Ag_3_PO_4_@UMOFNs photocatalyst, and the results indicated that the coupling system had a higher degradation efficiency (degradation rate increased to 1.6 and 1.8 times, respectively, compared with Ag_3_PO_4_) [58]. This result can be explained through the core–shell structure, where the separation efficiency of photogenerated electrons and holes as well as the response strength of the photocurrent can be significantly improved by a larger contact area, which was excited to produce more positive-charged holes (h^+^) and radicals for the photocatalytic reaction. Furthermore, the activity of Ag_3_PO_4_@UMOFNs increased because of synergistic effects.

### 3.6. Microorganisms

Within the rapid development in the global economy and the gradual increase in the urban population density, more and more harmful microorganisms (bacteria and fungi) are causing significant spread, which are large threats to human health [74]. It is well established that the abundant nanopore channels in MOFs facilitate the efficient encapsulation and delivery of bacterial substances. In contrast to monometallic MOFs, it is well known that BMOFs can provide tunable electron acceptance and energy-giving processes and generate different catalytically active sites and adsorption sites by adjusting the type and ratio of the two metal nodes [75]. Therefore, considering these advantages mentioned above, it is possible to employ BMOFs to enhance the antimicrobial capacity.

Tong *et al*. reported a kind of Co_x_Cu_y_-CAT-1 for *Escherichia coli* by synergistic photocatalytic–photothermal effect [59]. Co_x_Cu_y_-CAT-1 showed strong light absorption, low compound efficiency of photogenerated carriers and high photothermal conversion efficiency owing to coordination of Co and Cu. Besides, through the oxidation of ·OH radicals, a stable bacterial inhibition rate and efficient mineralization can be achieved, with a final total organic carbon (TOC) removal rate of about 70%.

Therefore, BMOFs and their based materials can be successfully applied to remove ions, dyes, PPCPs, phenolic compounds and microorganisms from wastewater sources by adsorption, oxidation activation, photocatalysis or photocatalytic–photothermal effect.

## 4. Pollutants removal mechanisms

**Figure 6.**
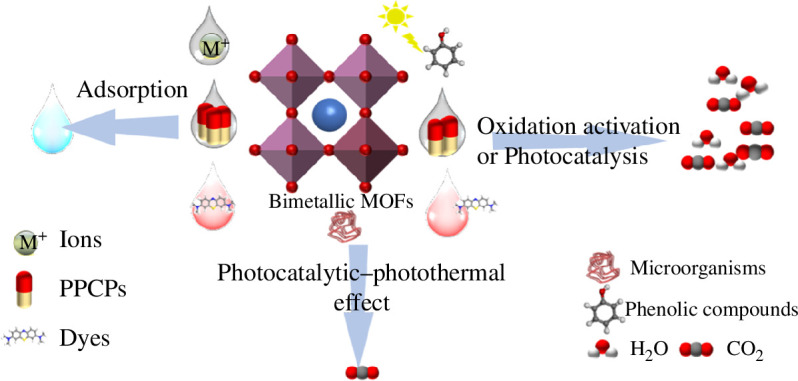
Schematic diagram of BMOFs for the removal of different pollutants.

We can conclude from the results in table 1 that BMOFs have a great removal ability of various pollutants in the water. That makes them a suitable weapon to deal with those harmful substances. Specifically, four suggesting mechanisms, adsorption, oxidative activation reaction, photocatalytic degradation and photocatalytic–photothermal effect, were involved in removing contaminants by BMOFs. Figure 6 shows a schematic diagram for the proposed mechanisms of pollutants. It illustrates the possible reactions on the surface of BMOFs with pollutants.

### 4.1. Adsorption

**Figure 7.**
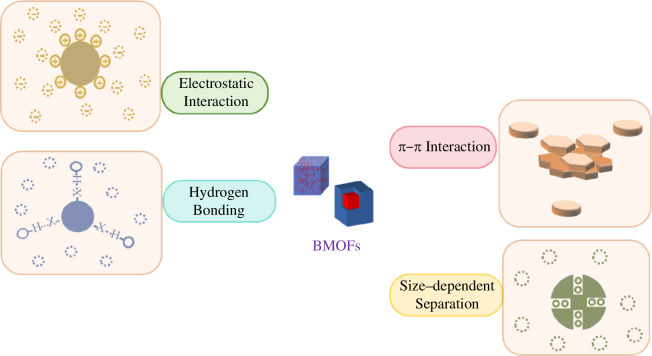
The possible mechanism of pollutants adsorption by BMOFs.

Adsorption of pollutants on BMOFs is one of the main processes for pollutants removal. Generally, pore structure, charge interactions between adsorbent and adsorbate and open metal sites in MOFs directly affect the adsorption behaviour [76,77]. Adsorption mechanisms mainly include physical adsorption, electrostatic attraction, hydrogen bonding between the adsorbent and the contaminant, π–π interactions, and so forth [78]. Figure 7 shows a schematic diagram for the proposed mechanism of the adsorption process.

In contrast to monometallic MOF-based materials, BMOFs have a larger surface area, richer pore structure and more available metal sites. Moreover, the synergistic effect of two active metal centres can enable BMOFs to exhibit excellent performance in terms of ultrafast adsorption kinetics and high adsorption capacity [65,66].

### 4.2. Oxidation activation

Undoubtedly, oxidation activation plays an irreplaceable role in the successful applications of water treatment. The Fenton reaction is the process of complete mineralization of organic pollutants by reactive species hydroxyl radical (·OH) generated by the interaction of ferrous ions (Fe^2+^) catalyst with hydrogen peroxide (H_2_O_2_) [79]. Unfortunately, in the traditional Fenton process, there are some drawbacks such as the need for strict pH regulation (pH 2.8–3.5) and sludge generation, which limit its application [80]. To deal with these problems, heterogeneous Fenton-like processes using solid catalysts is a good option. In the heterogeneous Fenton-like processes, instead of iron ions, other transition metal ions (e.g. cobalt, manganese, nickel and copper) are used as catalysts for the catalytic degradation of organic pollutants through the generation of ·OH by interacting with H_2_O_2_, which is similar in principle to the Fenton reaction [81]. BMOFs provide a high surface area and porous channels for the rapid transfer and diffusion of pollutants in the framework. In the presence of H_2_O_2_, BMOFs have a promising application as catalysts in the heterogeneous Fenton processes.

Liang *et al*. reported the catalysis in the degradation of ciprofloxacin (CIP) using MIL-101(Fe, Co) [29]. They found that after adding H_2_O_2_ in the system, Fe/Co bimetals as Lewis acid sites were conducive to the adsorption of H_2_O_2_ (as Lewis base) onto the active surface (equations (4.1) and (4.2)). Then, electron transfer from the electron-rich centres to H_2_O_2_ could effectively activate H_2_O_2_ to generate abundant ·OH radicals (equations (4.3)–(4.9)). In principle, the non-homogeneous Fenton reaction is similar to the homogeneous Fenton reaction, except that the oxidizing radicals in the former are generated at the catalyst surface, whereas the oxidizing radicals in the latter are generated from the solution [82].


(4.1) $$\equiv Fe\left(III\right)+H_{2}O_{2}\to \equiv Fe\left(III\right)-H_{2}O_{2}$$

(4.2) $$\equiv Co\left(II\right)+H_{2}O_{2}\to \equiv Co\left(II\right)-H_{2}O_{2}$$

(4.3) $$\equiv Fe\left(III\right)-H_{2}O_{2}\to \equiv Fe\left(II\right)+HO_{2}\cdot +H^{+}$$

(4.4) $$\equiv Fe\left(III\right)+HO_{2}\cdot \to \equiv Fe\left(II\right)+O_{2}+H^{+}$$

(4.5) $$\equiv Co\left(II\right)-H_{2}O_{2}\to \equiv Co\left(III\right)+\cdot OH+OH$$

(4.6) $$\equiv Fe\left(II\right)+H_{2}O_{2}\to \equiv Fe\left(III\right)+\cdot OH+OH^{-}$$

(4.7) $$\equiv Fe\left(III\right)+e^{-}\to \equiv Fe\left(II\right)$$

(4.8) $$\equiv Co\left(III\right)+e^{-}\to \equiv Co\left(II\right)$$

(4.9) ⋅OH+CIP→degradation products

The technology based on persulfate activation is gradually applied as a new technology to wastewater treatment because of their degradation and mineralization ability of difficult-to-degrade organic pollutants. As a multi-purpose material, BMOFs can generate high activity of free radicals (sulfate radical (SO_4_^−^), hydroxyl radical (·OH), superoxide radical (·O_2_^−^)) and singlet oxygen (^1^O_2_) by activating persulfate (peroxymonosulfate (PMS) and peroxydisulfate (PDS)) [83]. Zhang *et al*. used MIL-53(Fe, Ni) to activate PDS to degrade rhodamine B (RhB) with a removal efficiency of 93.9% within 180 min [26]. The following mechanisms were proposed. First, the high specific surface area and fully exposed active sites of MIL-53(Fe, Ni) provided diffusion pathways for contaminants, resulting in the adsorption of a large number of RhB molecules. Second, PDS was activated at the active site of MIL-53(Fe, Ni) to generate SO_4_^−^, ·OH and ·O_2_^−^ radicals (equations (4.10)–(4.12)). Third, low crystallinity promoted the generation of ^1^O_2_ (equation 4.13). Finally, the interconversion between PDS and metal ions drived the catalytic reaction all the way through (equations (4.14)–(4.16)). As a matter of fact, both free radicals (SO_4_^−^, ·OH and ·O_2_^−^) and singlet oxygen (^1^O_2_) were involved in the degradation of RhB (equation (4.17)).


(4.10) $$S_{2}O_{8}^{2-}+2H_{2}O\to HO_{2}^{-}+2SO_{4}^{2-}+3H^{+}$$

(4.11) $$S_{2}O_{8}^{2-}+HO_{2}^{-}\to SO_{4}^{2-}+SO_{4}^{\cdot -}+O_{2}^{\cdot -}+H^{+}$$

(4.12) $$SO_{4}^{\cdot -}+OH^{-}\to SO_{4}^{2-}+\cdot OH$$

(4.13) $$SO_{4}^{\cdot -}+H_{2}O+O_{2}\to \cdot OH+H^{+}{+}^{1}O_{2}+SO_{4}^{2-}$$

(4.14) $$S_{2}O_{8}^{2-}+M\left(II\right)\to 2SO_{4}^{\cdot -}+M\left(III\right)$$

(4.15) $$SO_{4}^{\cdot -}+SO_{4}^{\cdot -}\to S_{2}O_{8}^{2-}$$

(4.16) $$M\left(III\right)+e^{-}\to M\left(II\right)$$

(4.17) $$RhB+SO_{4}^{\cdot -}/\cdot OH/\cdot O_{2}^{-}{/}^{1}O_{2}\to \text{intermediate}\to \text{\textbackslash{} }CO_{2}+H_{2}O+\text{non-metal ions}.$$

### 4.3. Photocatalysis

Apart from the above mechanisms, photocatalysis is also a kind of important mechanism for removing contaminants [84,85]. Among the various types of reported photocatalysts, BMOFs show great potential in photocatalysis. Recent progress has shown that they can enable the exposed surface atoms to act as reaction sites owing to their large surface area and enhance their photocatalytic efficiency. Also, they show a high charge separation and a low carrier recombination rate. Besides, through the difference in electronegativity of the two metal ions, their electronic structures and photoresponses can be easily modulated to significantly improve the physicochemical properties of catalysts. The photocatalytic activity of BMOFs is induced by electron charge transfer from photoexcited organic ligands to metals within their internal structure [56]. During the design of BMOF-based catalysts, the two metal ions act in different roles, i.e. as catalytic centres and maintaining the stability of the catalyst structure, respectively. This design also allows for the isolation and stabilization of the active metal sites to achieve high activity, selectivity and stability [86].

**Figure 8.**
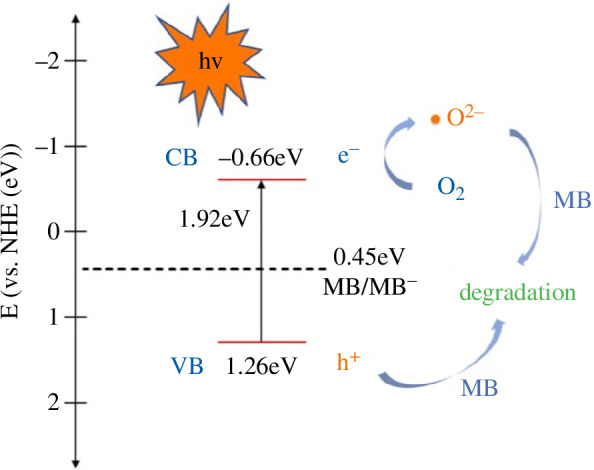
A schematic diagram of HNU-29 photocatalytic degradation of MB under visible light [20].

Feng *et al*. used HNU-29 to degrade MB with a removal efficiency of 92% under visible light [20]. The following mechanisms were proposed: when HNU−29 was irradiated with photons, electrons were excited from the valence band (VB) to the conduction band (CB) and formed h^+^ in the VB that mediated the degradation of MB molecules. Besides, electrons reacting with oxygen can form ·O_2_^−^, which showed a strong oxidizing ability for MB molecules (figure 8).

### 4.4. Photocatalytic–photothermal effect

Although photocatalysis has been considered as an effective mechanism for removing pollutants, there are still some limitations that prohibit its further applications, such as wide bandgap energies, low light absorption capacity and fast recombination rates of the photo-induced electrons and holes. On the basis of that fact, researchers proposed the photocatalytic–photothermal effect, which can achieve the more efficient use of sunlight as well as overcome the bottlenecks of photocatalysis [87]. Recently, some studies have focused on the synergistic effect of photocatalytic and photothermal processes using the solar light irradiation [88,89].

**Figure 9.**
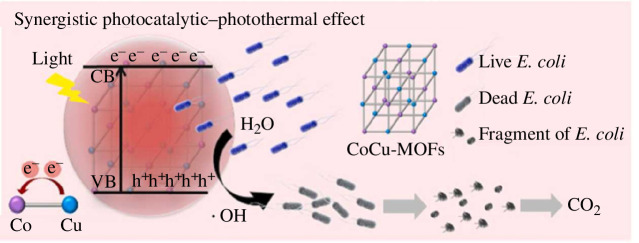
Illustration of different antibacterial processes on CoCu-MOFs [59].

In the field of antimicrobials, MOF has been considered to be a third-generation antimicrobial agent with broad application prospects. Tong *et al*. elaborated on the mechanism of antimicrobial activity of BMOFs: bacteria were oxidized on the surface of MOFs by photo-induced holes. Compared with single photocatalysis, which had limited light utilization, the synergistic photocatalytic–photothermal process made an induction to the more productive separation of charges. Therefore, more ·OH radicals had been produced by the reaction between photo-induced holes and H_2_O (figure 9). As a consequence, the ·OH radicals in the aqueous phase can successfully react with bacteria while overcoming the obstacles of surface coverage [59].

### 4.5. Theoretical calculations

**Figure 10.**
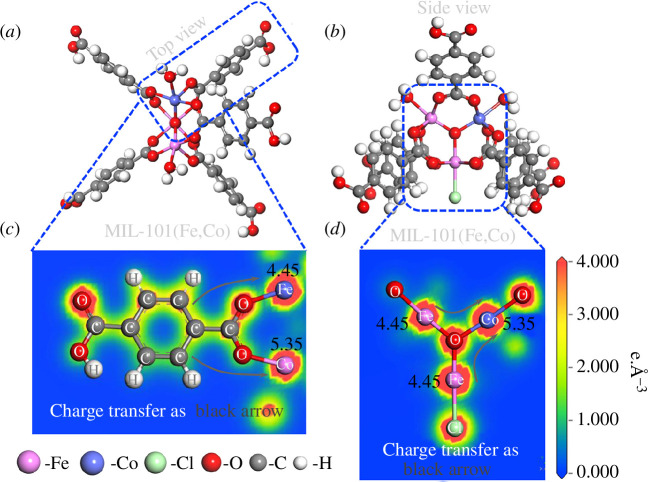
DFT calculations for the structure of different views (*a,b*) and the corresponding two-dimensional VED colour-filled maps (*c,d*) of the bimetallic MIL-101(Fe,Co). Grey, white, red, green, magenta and blue circles denote C, H, O, Cl, Fe and Co atoms, respectively [29].

In order to explore the mechanism of organic pollutants removal by BMOF materials thoroughly, a series of theoretical calculations were performed. Liang *et al*. investigated for the first time the effect of Co substitution on the electronic structure of MIL-101(Fe) by using density functional theory (DFT) calculations (figure 10) [29]. For the original MIL-101(Fe), the two-dimensional valence electron density (VED) surrounded by the benzene ring was lower than that surrounded by the oxygen and iron atoms. The result showed that π electrons tended to be transferred from the benzene ring to the transition metal centres via C–O–Fe bonds. Since the electronegativity of Co (1.88) was greater than the electronegativity of Fe (1.83), the introduction of Co atoms into MIL-101(Fe) caused the VED around Fe to decrease, which promoted the transfer of electrons from the Fe centre to the Co centre via the Fe–O–Co bonds, resulting in a maximum VED of 5.35 e·Å^−3^ for Co atoms in MIL-101(Fe,Co). These studies indicated the changed charge distribution in MIL-101(Fe,Co) caused by the construction of a dual site. As a result, electron-deficient centres were formed surrounded by the benzene rings, while the electron-rich centres were located near the atoms of Fe and Co. These calculations demonstrated that dual metal sites construction contributed to a better reaction with organic pollutants.

Zhang *et al*. predicted the reaction sites of RhB using the Fukui function [26]. Fukui functions had the following three manifestations:


(4.18) $$f^{-}\left(r\right)=\rho_{N}\left(r\right)-\rho_{N-1}\left(r\right)\approx \rho^{\text{HOMO}}\left(r\right)$$

(4.19) $$f^{+}\left(r\right)=\rho_{N+1}\left(r\right)-\rho_{N}\left(r\right)\approx \rho^{\text{LOMO}}\left(r\right)$$

(4.20) $$f^{0}\left(r\right)=\left[f^{+}\left(r\right)+f^{-}\left(r\right)\right]/2\approx \left[\rho^{\text{HOMO}}\left(r\right)+\rho^{\text{LOMO}}\left(r\right)\right]/2$$

**Figure 11.**
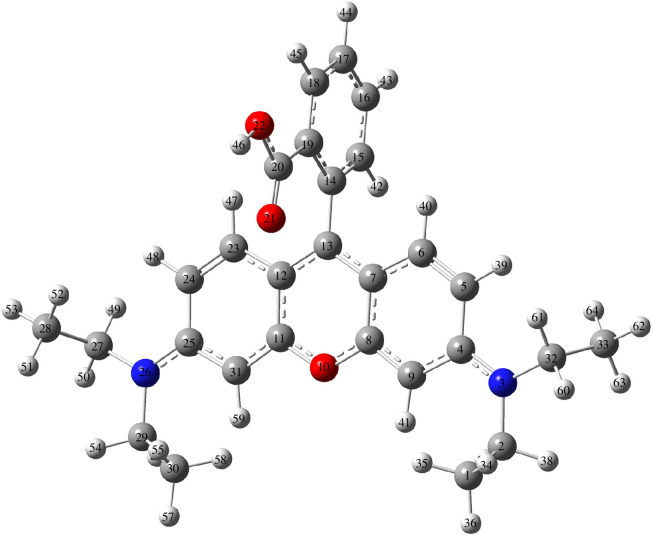
The optimized molecular structure of RhB [26].

In general, larger values represent a higher corresponding reactivity. Through the calculations, the authors found that the electron-rich positions of the RhB molecule were situated mainly at the O21 (the O atom number was 21, the same meaning as below), O22, C33, C30, C28, C1 and C9 atoms (figure 11).

**Figure 12.**
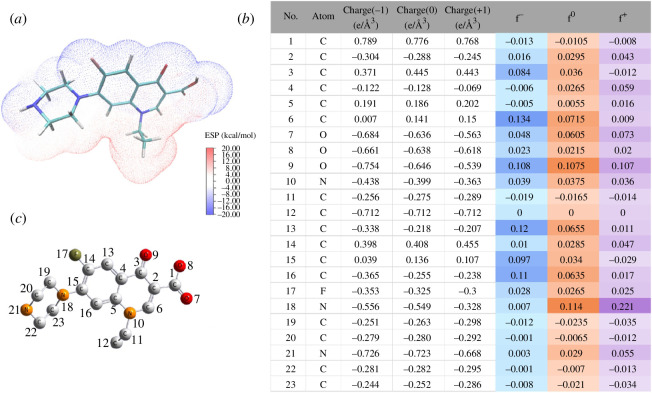
Electrostatic potential (ESP) map (*a*); Fukui index (*b*) and the detailed atomic position of NOR (*c*) [31].

Zhou’s group also predicted the reaction mechanism of NOR when it reacted with BMOFs through the Fukui function [31]. Based on the charge distribution of the Fukui index on nucleophilic (*f* ^−^) and electrophilic attacks (*f* ^+^), the most active sites were distributed in C6 (*f* ^◦^ = 0.0715), C13 (*f* ^◦^ = 0.0655), C16 (*f* ^◦^ = 0.0635), N18 (*f* ^◦^ = 0.114), N21 (*f* ^◦^ = 0.029) and N10 (*f* ^◦^ = 0.0375). These sites can be attacked preferentially to generate intermediate products to drive photocatalysis degradations. Moreover, the electrostatic potential (ESP) mapping of NOR indicated that blue atoms with high *f* ^◦^ values represented the reactive sites (figure 12).

In conclusion, BMOFs are used as either adsorbents or catalysts for pollutants. The reaction between BMOFs and pollutants generally includes three steps, i.e. (i) enrichment of pollutants on the surface of BMOFs by adsorption, (ii) reaction on the surface of BMOFs as a catalyst, and (iii) generation of strong oxidizing ions or free radicals to chemically degrade pollutants. Generally speaking, SO_4_^·−^, ·OH and ·O_2_^−^ radicals and ^1^O_2_ are the predominantly acting active species in the reaction.

## 5. Conclusion and outlook

In summary, BMOFs have attracted wide attention in the treatment of water pollution as a result of high porosity, diverse compositions, tunable pore structure and synergistic effects. BMOFs can form solid solutions or core–shell structures. Currently, the main ways to prepare BMOFs are one-pot method, post-synthesis modification method, template synthesis method, and so forth. Different preparation methods will strongly affect their morphology, structure and properties. Furthermore, owing to the synergistic effect between the bimetals and the unique porous structure, the BMOFs can effectively remove different kinds of pollutants, such as ions, dyes, PPCPs, phenolic compounds and microorganisms. The removal mechanisms mainly include adsorption, oxidative activation, photocatalysis and photocatalytic–photothermal effect. All these examples show that constructing BMOF-based wastewater treatment strategies is feasible and promising.

However, there remain some problems with the application of BMOFs in wastewater treatment to be addressed. First, the preparation methods may lead to problems, such as structural instability owing to the preparation method, difficult control of pore structure, uneven distribution of metal sites and leaching of metal ions. Hence, it is necessary to optimize the preparation of BMOFs by template-assisted enhancement of stability or adjustment of the void structure. Second, the preparations of some of these catalysts require the excessive use of chemicals, which are costly, complex and time consuming. Therefore, more economical and easier methods for BMOFs still need to be developed in the future. Third, some of the intermediate products produced during the catalytic process may be more toxic than those of the mother pollutants, leading to greater harm to the environment and human beings. As a result, it is desirable to carry out the extensive toxicological studies of the intermediate products. Finally, for better removal results, researchers can construct novel BMOF-based catalysts by compounding with inorganic semiconductor materials. Moreover, adding biological systems such as membrane bioreactors is also a good option.

Overall, owing to the high porosity, diverse compositions, tunable pore structure and synergistic effects, BMOFs will find more environmental uses in the near future. By reviewing their preparation methods and applications in water treatment, we hope that this review can assist researchers to better understand BMOFs for removing pollutants in water.

## Data Availability

This article has no additional data.
